# Exploring the Operative Strategy for Secondary Mitral Regurgitation: A Systematic Review

**DOI:** 10.1155/2021/3466813

**Published:** 2021-06-22

**Authors:** Francesco Nappi, Sanjeet Singh Avtaar Singh, Francesca Bellomo, Pierluigi Nappi, Camilla Chello, Adelaide Iervolino, Massimo Chello

**Affiliations:** ^1^Department of Cardiac Surgery, Centre Cardiologique du Nord de Saint-Denis, Paris, France; ^2^Department of Cardiothoracic Surgery, Golden Jubilee National Hospital, Glasgow, UK; ^3^Department of Clinical and Experimental Medicine, University of Messina, Italy; ^4^Regenerative Medicine, Università Campus Bio-Medico di Roma, 00128 Rome, Italy; ^5^Department of Cardiovascular Sciences, Fondazione Policlinico Universitario A. Gemelli IRCSS, Italy; ^6^Cardiovascular Surgery, Università Campus Bio-Medico di Roma, 00128 Rome, Italy

## Abstract

**Background:**

Mitral valve disease surgery is an evolving field with multiple possible interventions. There is an increasing body of evidence regarding the optimal strategy in secondary mitral regurgitation where the pathology lies within the ventricle. We conducted a systematic review to identify the benefits and limitations of each surgical option.

**Methods:**

A systematic review of the literature was performed to identify pertinent randomized controlled trials (RCTs), propensity-matched observational series, and meta-analyses which were considered initially and followed by unmatched observational series using the MEDLINE, Ovid EMBASE, and Cochrane Library.

**Results:**

We identified 6 different strategies for treating secondary mitral valve regurgitation: mitral valve replacement, restrictive mitral annuloplasty, surgical revascularization (with and without mitral annuloplasty), subvalvular procedures (papillary muscle approximation, papillary muscle relocation, ring and string procedure), and procedures directly targeting the mitral valve (edge-to-edge repair and anterior leaflet enlargement) alongside transcatheter heart valve therapy. We also highlighted the role of left ventricular assist devices in the management of this condition. The benefits and limitations of each intervention are highlighted.

**Conclusion:**

There is currently no unanimous and shared strategy for the optimal treatment of patients with secondary IMR. The management of patients with secondary mitral regurgitation must be entrusted to a multidisciplinary Heart Team to ensure ideal intervention and patient matching for the best outcomes.

## 1. Introduction

Following the recommendations of the current guidelines, the use of mitral valve replacement (MVR) for secondary mitral regurgitation (S-MVR) has been widely adopted by the surgical community, and the vast majority of patients currently receive valve-sparing surgery to replace the ischemic mitral valve [1, 2]. In the last 5 years, we have witnessed a progressive decline of the mitral valve repair using restrictive annuloplasty (RMA), which had been established in the 80s following the recommended indications of the French correction [3]. On the other hand, the advent of the MitraClip procedure in extending the possible mechanical intervention procedures is clouded by a veil of uncertainty due to conflicting evidence in supporting the benefit in terms of efficacy and safety [4, 5].

Resistance among surgeons to the use of subvalvular repair (SR) combined with RMA can be explained, at least in part, by the fact that clinical benefit of additional handling the subvalvular apparatus which has been reported in observational studies [6–8] has been confirmed in only a few randomized clinical trials [9, 10]. Although these randomized studies have shown few rates of mitral regurgitation recurrence and reoperation with the combined procedure using surgery of papillary muscles (PMs), these trials were individually underpowered to detect differences in the frequency of clinical events. Therefore, whether the use of subvalvular repair can improve clinical outcomes over time remains unknown. Moreover, there is a clear contradiction between the proven benefits of handling the PMs and their very limited use in everyday clinical practice.

## 2. Methods

### 2.1. Eligibility Criteria

We systematically reviewed the literature to identify pertinent randomized controlled trials (RCTs), propensity-matched observational series, and meta-analyses that were considered initially and followed by unmatched observational series.

The criteria for considering the eligibility and inclusion of manuscripts in the study were patients aged ≥ 18 years and the mechanical intervention including mitral valve replacement, repair, and transcatheter mitral valve therapy.

Exclusions are consisted of studies involving animal or pediatric studies and nonprimary studies (i.e., letters, editorials, and review articles). Another exclusion criterion for manuscripts included the inability to obtain a translation or when articles were published only as abstracts.

### 2.2. Search Strategy

The search was carried out on February 1, 2021, using the following databases: Ovid MEDLINE (1946 to the present), Ovid EMBASE (1947 to the present), and the Cochrane Library (Wiley; 1956 to the present).

### 2.3. Data Extraction

**Figure 1:**
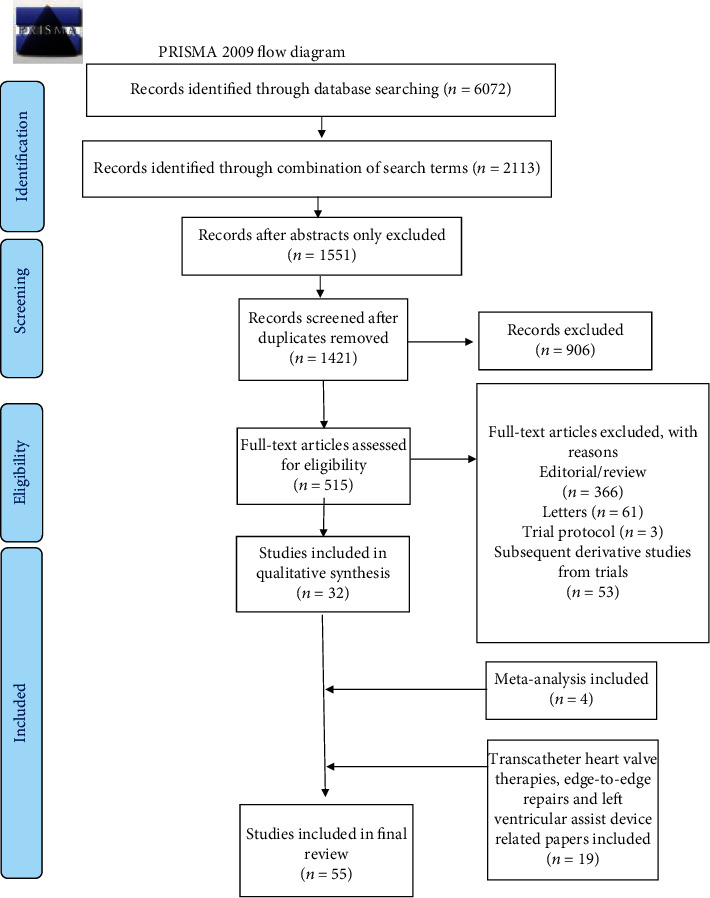
The PRISMA Chart.

Three investigators (SSAS, FB, and CC) searched papers published between January 1, 1987, to present using the following terms: “secondary mitral regurgitation,” “ischemic mitral regurgitation,” and “functional mitral regurgitation.” These terms were coupled with « mitral valve replacement », « mitral valve repair », “restrictive mitral annuloplasty,” and “transcatheter heart valve implantation.” For completion, the following terms were added: subvalvular repair, papillary muscle approximation, papillary muscle relocation, and papillary muscle sling. Randomized controlled trials (RCT), propensity-matched observational series, and meta-analyses were considered initially and followed by unmatched observational series. Pertinent abstracts were reviewed, and data were extracted independently for all enclosed manuscripts. The correct progression of the study was verified by a fourth investigator (F.N.) to ensure accuracy. The PRISMA Chart and PRISMA 2020 Checklist Item are enclosed in Figure 1 and Table 1.

Extracted data were transposed onto a table with the following subheadings: study completion date, number of patients, follow-up period, primary and secondary end points, and study findings.

### 2.4. Risk of Bias Assessment

The Cochrane Risk of Bias assessment tool was used for randomized controlled trials. Assessment of bias of the RCTs showed a low risk of reporting, detection, or attrition bias as the outcomes were well reported and the loss to follow-up was low.

Given significant heterogeneity in study design across identified studies, formal data synthesis via meta-analysis was not conducted. Hazard Ratios (HR) with 95% CIs were calculated where possible for primary and secondary endpoints as shown in Tables 1–5.

## 3. Results

This was the largest series of mitral valve replacement and repair operations reported which considered the various mitral valve repair techniques, the reported results for each procedure, and evaluating indications, clinical uses, clinical evidence, and limitations. Follow-up of patients with severe left ventricular function dysfunction for which the use of a left ventricular assistance device (LVAD) was indicated was also included.

## 4. Mitral Valve Replacement

### 4.1. Indication

In the past few years, the use of mitral valve repair has greatly exceeded the use of replacement. However, recently, a randomized trial established the superiority of replacement across a spectrum of patients with severe ischemic mitral regurgitation [1, 2]. The survival benefits associated with the use of chordal sparing mitral valve replacement (MVR) were established in a landmark paper from David et al. [11] almost 40 years ago in which the authors documented that the continuity between mitral annulus, left ventricular wall through leaflets, chordae tendinae, and papillary muscles play a pivotal role in left ventricular function after mitral valve surgery. Table 1 highlights the studies reporting the use of MV replacement.

### 4.2. Clinical Use

The improved outcome using the total chordal preservation surgery compared to posterior leaflet only is almost certainly due to its superior long-term reduced left ventricular chamber size and systolic afterload [12]. Individual studies have reported several disadvantages in patients who underwent partial chordal preservation compared to those who received mitral valve repair including higher operative mortality, improved left ventricular function, and higher rates of long-term survival [13–15]. Its peculiar morphologic features probably explain the superior efficacy of the total chordal sparing preservation in determining the higher improvement of wall motion in the apical and diaphragmatic region of the left ventricle [16]. The Stanford group [12] showed that although a decrease of left end-diastolic volume (LVEDV) has been noted both in recipients of total (176 ± 36 vs. 118 ± 24; mean change from baseline −58 ± 12; *P*.0001) and partial chordal sparing mitral valve replacement (145 ± 57 vs. 114 ± 52; mean change from baseline −31 ± 5; *P*.0001) at postoperative discharge, preservation of the entire subvalvular apparatus may determine a continuous and progressive reduction of LVEDV that was noted to have fallen further only after 1 year compared to partial preservation (112 ± 41 vs. 107 ± 28; mean change from baseline −69 ± 8 vs. 33 ± 16; *P* = 0.63). Moreover, compared to partial preservation, the left ventricular end-systolic volume (LVESV) continued to decline over time in patients who received total chordal preservation (60 ± 13 vs. 40 ± 11; mean change from baseline −20 ± 2; *P*.0001), but no notable changes were observed in recipients of partial chordal sparing (46 ± 24 vs. 50 ± 20; mean change from baseline 4 ± 4; *P*.0.9).

### 4.3. Clinical Evidence

Only 1 published RCT has compared outcomes between complete preservation of all chordal structures and mitral valve repair. The CTSN trial (Cardiothoracic Surgical Trials Network) recruited 251 patients in 23 countries. The primary outcome was the degree of left ventricular reverse remodeling, which was defined as the LVESV at 1 year after randomization. An interim analysis at 2 years reported no significant between-group difference of left ventricular reverse remodeling (60.6 ± 39.0 ml in chordal sparing MVR vs. 52.6 ± 27.7 in MV repair; mean change from baseline, −6.5 ml and −9.0 ml, respectively) [2].

While awaiting the outcome of the CTSN trial at more than 2 years, there is currently a substantial body of exhaustive evidence to support the use of chordal sparing MV replacement operation, as it appears to offer an additional benefit on mitral regurgitation recurrence over the mitral valve repair [17, 18]. On the other hand, almost 10 years ago, a systematic review of matched cohorts of 600 patients who received MV repair (*n* = 416) or MV replacement (*n* = 184) reported a long-term risk of death that was 35% higher in the replacement group than in the repair group (hazard ratio 1.352 (95% CI 1.131–1.618)) [19].

In the past 20 years, 2 largely independent studies have supported any definitive conclusions about the relative effects of the two surgical procedures on survival. One study included 370 patients and reported an overall 6-year survival that was not statistically different between MV repair and MV replacement (73 ± 4% versus 67 ± 4%; *P* = 0.17; HR, 1.2; 95% CI, 0.7–1.9; *P* = 0.52) [20]. Another study included 1250 patients, with follow-up duration exceeding a mean of 5 years, and reported a survival at 10 years of 36% in patients who underwent MV replacement and 33% in those who received restrictive mitral valve repair, with no significant difference between groups in patients older than 60 years (*P* = 0.34). The survival benefit of chordal sparing operations was less evident in patients younger than 60 years of age. In recipients of mitral repair, the survival was 81% compared with 55% for those who received valve replacement (*P* = 0.0001) [21].

### 4.4. Limitation

The major concern with the use of mitral valve replacement without preservation of valve apparatus during the surgery of IMR is the increased risk of reduced postoperative ejection performance. This pathophysiological condition has been associated with the rapid increase in systolic wall stress leading to the elimination of the low-impedance pathway for ejection into the left atrium and to ventricular dysfunction caused by breaking of the mitral valve apparatus.

Rozich et al. [22] showed that a mitral replacement with chordal preservation significantly decreases the incidence of reduced LV end-diastolic and end-systolic volumes. End-systolic wall stress decreased from 95 ± 6 to 66 ± 6 g/cm^2^ (*P* < 0.05) in the chordal preservation group and increased from 89 ± 9 to 111 ± +12 g/cm^2^ (*P* < 0.05) in the chordal transection group. Ejection fraction was unchanged before and after mitral valve surgery (from 0.63 ± 0.01 to 0.61 ± 0.02) in patients who received chordal sparing operation instead showing a marked reduction in those without the use of chordal sparing (from 0.60 ± 0.02 to 36 ± 0.02).

However, the incidence of serious ventricular function problems can be significantly reduced by well-advised patient selection and choice of surgical technique for IMR correction. In young patients, consideration should be given to avoiding total chordal sparing mitral valve replacement in favor of RMA with subvalvular repair if they have preoperatively echocardiographic asymmetric leaflet tethering, posterior leaflet tethering angle of <45°, tenting height < 11 mm, absence of a basal aneurysm/dyskinesis, no greater degree of LV dilation (LVEDD < 60 mmHg), and LV sphericity index [23]. Moreover, Michler reported that MV tenting area larger than 3.1 cm^2^, rather than LVEDD greater than 64 mm and LVESD greater than 54 mm, significantly reduces the risk of persistent or recurrent MR when chordal-sparing MV replacement is used [24].

## 5. Restrictive Mitral Annuloplasty

### 5.1. Theory and Indication

Introduced in mitral surgery in the 1980s [3], the use of restrictive mitral annuloplasty has been widely adopted by the surgical community in patients with moderate to severe ischemic mitral regurgitation. The ischemic MR is prevalently coupled with regional inferior wall motion abnormalities, leading to posterior leaflet tethering and posteriorly directed mitral regurgitation (Carpentier type IIIb). Bolling and Bach [25, 26] firstly published the results from a large series of patients with end-stage cardiomyopathy who underwent mitral valve reconstruction.

The mechanism of restrictive mitral annuloplasty, by bringing the leaflets closer together, is to reduce the anteroposterior diameter to minimize the tenting area and favor normalization of the coaptation length. A consistent addition to appropriate-sized annular correction with RA is to reduce the LVES dimension or volume. Therefore, the abolition of MR and the decrease of the size of the left ventricle consequently favor reduced stress of the LV wall, inducing an improvement of LV reverse remodeling [27].

In patients with moderate IMR, the use of RMA combined with CABG remains a point of discussion. Penicka and colleagues demonstrated that improvements in global and regional wall motion, as well as reverse LV remodeling after CABG alone, are indicative of viable myocardium. In their series of patients with moderate IMR and who received CABG alone, the resolution of MR after surgery was associated with more viable segments and less LV dyssynchrony at baseline. The role of CABG and the presence of viable myocardium are emphasized in the results of the CTSN trial where 25% of patients did not undergo CABG operation thus eliminating the possibility of improved regional wall motion. Hence, the RMA was rendered ineffective leading to the inability of the leaflets to establish the appropriate zone of coaptation to reduce the tenting height and tenting area [1, 2].

Tables 2 and 3 are the studies reporting the use of MV replacement.

### 5.2. Clinical Use

The most widely used dogma in RMA is to downsize by 2 ring sizes (i.e., size 28 when measuring size 32). The overcorrection by undersizing MV repair and the technique using rigid versus partial bands or flexible complete rings affect MR recurrence rates. Although some evidence has shown that recurrent MR is more frequent in recipients of partial bands or flexible complete rings [28], recurrence rates of MV regurgitation remain high even in patients who received complete rigid rings [18, 28, 29]. Generally, RMA is carried out by downsizing 2 ring sizes if the LVEDD less than 60 mm and LVESD of less than 55 mm. In a small RCT, we described the use of a double row overlapping suture that has shown effectiveness in preventing MR recurrence at two years follow compared to the single row [30]. In ischemic MR patients, we always perform complete revascularization (Tables 2 and 3).

The concept of “the tighter, the better” tenaciously advocated by most surgeons [3, 9, 14, 23, 30, 31] for the RA overcorrection today seems to falter as we have a better comprehension of the impact of RA on the spatial relationship between the left ventricle and the mitral annulus [32]. The use of RMA virtually disturbs this ischemia-induced aberrant geometric spatial relationship further by exacerbating the tethering of the posterior leaflet. This phenomenon is supported by the persistently ischemic lateral and posterior papillary muscle displacement relative to the mitral annulus. Anatomically, the anterior leaflet is attached to the fibrous trigones with the consequence that the posterior leaflet is primarily affected by RMA with decreasing of the mitral annular area and reduction of the anterior-posterior dimension. In presence of a persistent displacement of the posterior-inferior-lateral wall of the LV, overcorrection may worsen the geometric mismatch between the left ventricle and the mitral annulus with an exacerbation of the posterior leaflet tethering. Suggested by these observations, the CTSN investigators and evaluating the ratio between LV end-systolic dimension (LVESD) and prosthetic annuloplasty ring size may predict the risk of persistent or recurrent MR. They have established that a ratio of LVESD to ring size of 2 or greater was associated with an increased risk of persistent or recurrent MR. Increased tethering between the papillary muscle and the leaflet edge may be produced by overcorrection of the annular dimension. The authors concluded that although RA is a necessary operation, it is a poor procedure for the treatment of severe IMR [ 32]. These concepts of pathophysiology open the way for a rigorous preoperative evaluation by surgeons who can easily prevent this problem and plan the best choice in the operating room accordingly [33–37] (Table 3).

### 5.3. Clinical Evidence

The largest echocardiographic series report an MR recurrence range from 15% of patients at 6 months to 56.8% of patients at 24 months [1, 2] and from 55% to 70% at 5-year follow-up [14, 23]. The severity of preoperative MR is a key factor in determining MR recurrence. There is general agreement that the MR recurrence should be manifested in patients with centrally directed or multiple jets, a greater degree of LV dilation, symmetric anterior leaflet tethering, presence of a basal aneurysm/dyskinesis, ≥11 mm coaptation height, and a posterior leaflet angle of >45° [28, 34–38].

Kron et al. [28] showed that patients with severe IMR who experience persistent recurrent MR after undersized MV repairs have poorer LVESV at 2-year follow-up compared to patients with no recurrent MR (45 ± 10 vs. 42 ± 10; *P*.097; mean change from baseline, −16.1 ml and – 19.1 ml, respectively). Similarly, Nappi et al. [34] found a worsening of LVESD in patients with moderate to severe MR recurrence at 5-year follow-up (55 vs. *X*; *P* = *Y*; mean change from baseline, − *X* ml and – *Y* ml, respectively).

The use of restrictive mitral annuloplasty alone is the valve repair operation for which there is the most evidence derived from individual studies. Several reports have compared the RMA with mitral valve replacement (Table 2). All studies that extended the follow-up beyond the five postoperative year showed significantly higher rates of mitral regurgitation recurrence for the undersizing mitral valve repair over the mitral valve replacement and a substantial similar rate of survival at longer follow-up. In 2 studies, 1 RCT [2] and 1 PMS [39], a tendency toward a higher incidence of clinical events was also found for RMA patients.

The Duke group [14] showed that trends in numbers of patients who received a restrictive mitral repair (*n* = 416) versus partial or total chordal sparing operation (*n* = 106) over a 20-year period (from 1986 to 2006) increased in later years (from 2000 to 2006). The use of MV repair vs. MV replacement seemed to be related to surgeon choice and preference highlighting that the judgment of the operating surgeons was by far the most important factor determining the choice of repair versus replacement (Wald *χ*2 = 58.9, *P* = 0.0001). Nevertheless, the severity of MR (Wald *χ*2 = 19.9, OR = 3.377, 95% Cl (1.977, 5.766), *P* = 0.0001) was a factor influencing the choice of surgeon with a higher percentage of MVR compared to RMA in patients with moderate to severe MR. Additionally, patients selected for replacement tended to have better ejection fractions (Wald *χ*2 = 11.4, OR = 1.277, CI 95% (1.108, 1.472), *P* = 0.0007) [14].

### 5.4. Limitation

**Figure 2:**
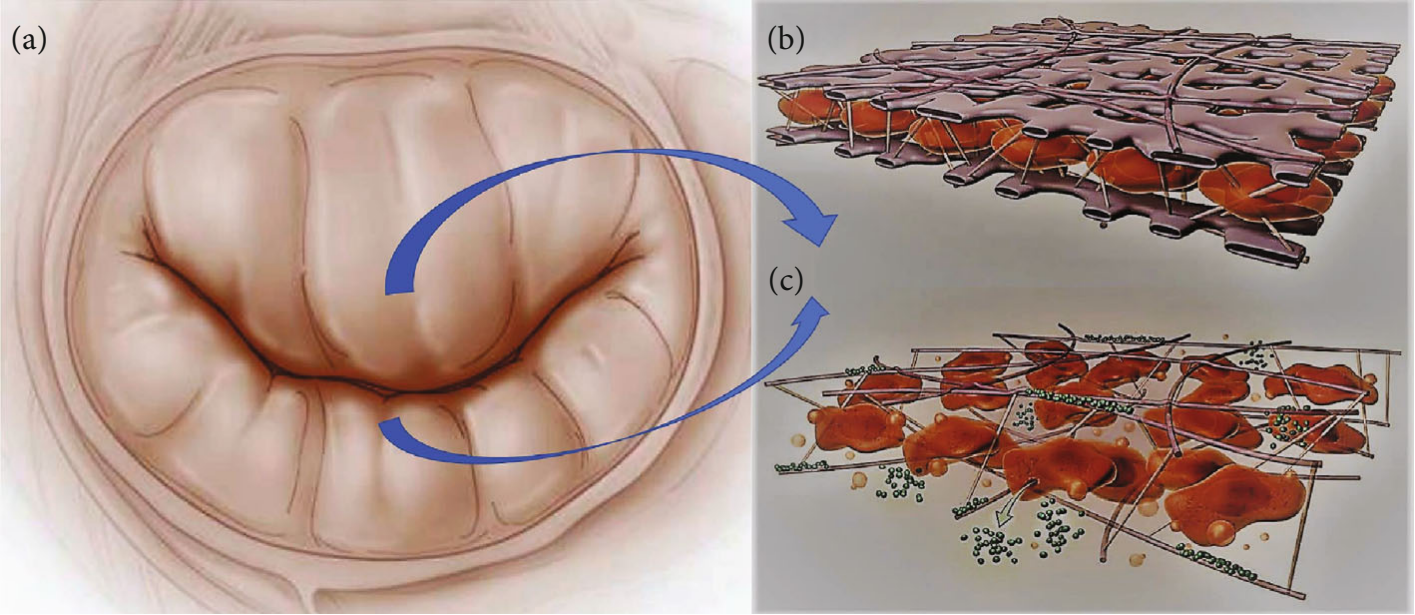
(a–c) Apoptotic and fibrotic change involving mitral leaflets in secondary mitral regurgitation. The harmful effect is due to leaflets tethering mediated by transforming growth factor- (TGF-) *β* secondary PM displacement and traction. (b) Absence of leaflet thickness without profibrotic changes of tethered MV leaflets leads to integrity of mitral leaflet structure. (c) Mitral valve leaflet fibrosis post-MI is associated with excessive endothelial-to-mesenchymal transition driven by TGF-*β* overexpression. Evidence with profibrotic changes due to tethering of MV leaflet post-MI.

The main limitation to RMA is the excessive geometric distortion of the left ventricle and an accentuating tethering of the leaflets with the unfavorable remodeling of mitral leaflets (Figure 2). Several studies have shown that regardless of whether 1 or 2 sizes is used to proceed with undersizing of RMA, the procedure is not recommended [2, 9, 10, 23]. A substantial contribution to understanding the limitation of RMA was made by the Osaka group [40–42]. Restrictive mitral annuloplasty causes a temporary improvement in mitral regurgitation when the LVEDD is less than 60 mm and LVESD is less than 55 mm. Patients with severe IMR who did not experience persistent or recurrent MR after RMA had a smaller left ventricle during a median follow-up period of 66 months compared to patients with recurrent MR after RMA alone. In addition, the LV function improved due to the reduced anterior and posterior PM tethering distance, anterior leaflet angle, and IPMD for 33 patients [40] Tables 2 and 3.

## 6. CABG and Moderate IMR

### 6.1. Theory and Indication

There is currently no single surgical management of moderate ischemic mitral regurgitation at the time of CABG which is based on a widely diffused consensus-oriented towards a single procedural direction. Two points of view are compared.

Some experts believe that revascularization alone for moderate ischemic mitral regurgitation can reduce rates of mitral regurgitation. The principle is related to the fact that there is an improvement in regional and global left ventricular function and geometry after CABG [43, 44].

The detractors of this choice support the use of RMA combined with CABG and therefore intervene directly on the MR. In this case, further unfavorable remodeling can be avoided, and the risk of heart failure decreased [45, 46].

### 6.2. Clinical Use

Observational studies addressing the differences in the MR recurrence of the 2 surgical strategies.

(CABG +/- RMA) and a nonsignificant tendency to better event-free survival for the RMA are discordant and usually have major methodological or sample-size limitations. The reliability of retrospective cohort studies, even if improved by a statistical analysis based on the propensity score, is bound by variables that are often not measurable. The most important is the surgeon's decision to consider with surety, a safety margin to permit restoration of the IMR after myocardial revascularization which leads to a favorable remodeling of the left ventricle [24, 44].

### 6.3. Clinical Evidence

Four recent randomized clinical trials [45–48] of moderate IMR with well-defined criteria for the measurement of MR have compared CABG alone versus CABG plus RMA. Bouchard et al. [48] showed that the addition of a ring did not change the clinical course after CABG surgery. At 12 months, no echocardiographic difference was found in terms of residual mitral regurgitation (*P* = 0.316), LVESV (*P* = 0.427), and left ventricular function (*P* = 0.204). The assessments of heart failure using the Minnesota QOL score and BNP measurements were not discriminatory of better improvement with either CABG alone or combined strategy.

In a study from the National Heart, Lung, and Blood Institute-sponsored Cardiothoracic Surgical Trials Network (CTSN) [47], CABG alone was compared with CABG and RMA in 301 consecutive patients who were followed for a maximum of 2 years. The patients who underwent a combined procedure (RMA group) resulted in a significant reduction in the rate of MR recurrence as compared with CABG alone at 1 and 2 years, with no progression to severe MR was noted in the RMA group (31.0% (moderate, 25.9%; severe, 5.2%) vs. 11.2% (moderate, 10.4%; severe, 0.8%); *P* < 0.001). There was no significant survival benefit for the patients who underwent combined CABG plus RMA as compared with those who CABG alone (2-year survival, 10% vs. 10.6%; HR in the combined-procedure group, 0.90; 95% confidence interval (CI), 0.45 to 1.83; *P* = 0.78). Likewise, there were no differences between the 2 groups in New York Heart Association functional class, LV ejection fraction, survival, or major adverse cardiac and cerebrovascular events at 2 years. The investigators noted that the RMA group had a longer hospital stay after surgery, a higher incidence of postoperative supraventricular arrhythmias during the first year (24 events vs. 11 events, *P* = 0.04), and more postoperative neurologic events (14 events vs. 4 events, *P* = 0.02), which included metabolic encephalopathy, seizures, transient ischemic attack, and stroke [43].

Both POINT and RIME (Randomized Ischemic Mitral Evaluation investigators) [45, 46] revealed that the association of RMA to CABG resulted in improvements in LV reverse remodeling, LV ejection fraction, New York Heart Association functional class, and MR grade but not in survival. POINT RCT [45] evaluated the effect of surgery on long-term outcomes in 102 patients that were randomly assigned to undergo CABG alone or CABG plus RMA. The 48 patients undergoing the combined RMA plus CABG significantly reduced LVEDD at five years follow than those whose moderate mitral regurgitation was managed with CABG alone (59 ± 8 vs. 52 ± 7 and 58 ± 7 vs. 56 ± 8; *P* < 0.001). In RIME trial [46], 73 patients with moderate IMR were studied, and 39 patients undergo CABG alone while 34 received CABG plus RMA. During a median follow-up period of 12 months, the recipients of combined surgery had a 28% reduction in LV end-systolic volume index (LVESVI) relative to baseline (78.4 ± 26.5 vs. 56.2 ± 14.9; mean baseline −22.2 ± 25.6 and 71.8 ± 16.1 vs. 67.4 ± 20.1 mean baseline -4.4 ± 17.4).

Two meta-analyses pooled data from RCTs, and large observational studies have compared the use of RMA plus CABG-to-CABG alone [49, 50]. Again, all studies with a mean follow-up time extending beyond the first postoperative year reported significant benefits in terms of prevention of MR recurrence when undersizing mitral annuloplasty was used (Table 3). One meta-analysis included 9 retrospective studies, and a total of 2479 patients with moderate to severe IMR (grades 2.2 to 3.9) undergoing CABG (*n* = 1515) or CABG combined with mitral valve surgery (*n* = 964). The authors reported no benefit for long-term survival (risk ratios 1.02; 95% confidence interval (CI) 0.90 to 1.14; *P* = 0.73); however, patients who had RMA decreased the MR grade more than those who underwent CABG-alone operation (standardized mean difference -0.9; 95% CI -1.250 to -0.559; *P* < 0.0001). NYHA class improvement was not statistically demonstrated (SMD = −0.26; 95% CI -0.766 to -0.24; *P* = 0.30) [49].

The only comparative meta-analysis of 4 RCTs and 15 observational studies (OS), with clinical and echocardiographic endpoints, has compared patients with moderate-to-severe IMR who received CABG alone and those who underwent CABG and mitral valve surgery (18 RMA and 5 MVR). The use of combined CABG and MVS was not associated with an increased perioperative mortality (RCTs: relative risk (RR) 0.89, 95% confidence interval (CI), 0.26-3.02; OS: RR 1.40, 95% CI, 0.88-2.23) but reduced cardiac events (myocardial infarction, heart failure, ischemia) for the RA (RR, 0.49; 95% CI, 0.28 to 0.87; *P* = 0.014). Patients who had coupled CABG and MV surgery showed a significantly lower incidence of moderate-to-severe MR at follow-up (RCTs: RR 0.16, 95% CI, 0.04-0.75; OS: RR 0.20, 95% CI, 0.09-0.48). Late mortality was similar between the surgical approaches in RCTs (hazard ratio (HR) 1.20, 95% CI, 0.57-2.53) and OS (HR 0.99, 95% CI, 0.81-1.21). There were no significant differences in echocardiographic outcomes [50].

### 6.4. Limitation

Although the use of undersized valve repair for MV surgery can augment severe ischemic mitral regurgitation, the surgery has never overtly demonstrated a change in the natural history of the main disease (dilated ischemic cardiomyopathy) or improve survival [14, 51]. Concerns over mitral regurgitation recurrence, due to an overcorrected restrictive mitral annuloplasty repair, have been shown after the demonstration of progressively augmented leaflet tethering caused by the anterior displacement of the posterior leaflet, as well as progressive adverse global and localized left ventricular remodeling [52–55]. This finding is probably the anatomic background for the demonstrated lack of utility of long-term surgical therapy in patients with undersizing MV repair [24, 33–35, 55–59] even though such strategy combined with CABG is still widely adopted in the surgical community in a patient with moderate IMR [44–48].

## 7. Subvalvular Procedures

**Figure 3:**
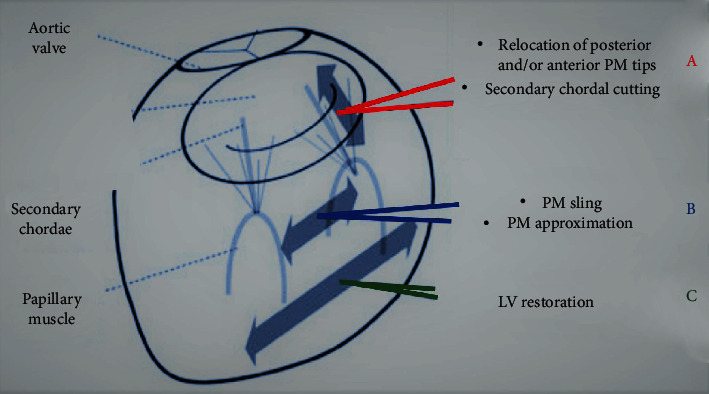
(a–c) The use of subvalvular repair is aimed at correcting the geometric alteration involving subvalvular apparatus. (a) PM relocation fixes the papillary muscle to the posterior and/or anterior trigone. The cutting secondary chordae reduces the tension exerted on the leaflets. (b) The PM approximation reduces the distance between the papillary muscles. (c) The subvalvular repair is effective in restoring correct ventricular geometry.

Subvalvular procedures are commonly used in addition to annuloplasty with the purpose to recondition both the functionality and configuration of the subvalvular apparatus. The main effect of subvalvular repair is the reduction of tethering forces exerted on both leaflets of MV due to lateral and posterior dislocation of PMs. Unlike the RMA which can indirectly influence the recovery of LV geometry through a favorable reverse remodeling, subvalvular repair has a targeted action in improving left ventricular geometric disorder. There are different surgical techniques for handling the subvalvular apparatus referring to peculiar concepts, and each procedure should be selected considering the direction of MV tethering (apical, lateral, or posterior). Here, we will discuss three techniques with possible variations: the papillary muscle approximation, the papillary muscle relocation, and the ring + string procedure [7, 60–62] (Table 5 and Figure 3).

### 7.1. Theory and Indication

The principle that strengthens the three main surgical techniques of handling the papillary muscles is the ability to reconfigure the correct geometry of the left ventricle that has been compromised by the vector displacement of the PMs (Figure 3(c)).

Indeed, the ischemic mitral regurgitation being a pathology that alters the geometry of the MV compromises three main dimensions: the anteroposterior diameter of the annulus, the tenting area, and the interpapillary muscle distance.

The objective in using the subvalvular correction procedure in association with RMA not only guarantees correction of the interpapillary muscle distance directly but allows monitoring of overcorrection through the annuloplasty, to develop mitral valve stenosis as complication [63, 64].

The concept of the role of the movement of PMs alongside three directions (apical, lateral, and posterior) has been accepted by the community of cardiologists and cardiac surgeons with the result of directing both the surgical indication and the choice of treatment [65, 66]. The most recent study by Stanford University has suggested posterolateral posterior papillary muscle displacement as the predominant pathomechanism leading to apical leaflet tethering during IMR [67].

#### 7.1.1. Papillary Muscle Approximation

*(1) Clinical Use*. Initially described by Hvass et al. [9], the rapprochement of PMs was performed using a 4 mm Goretex prosthesis. It was passed through the trabeculae of the ventricle, bringing the papillary muscles completely close. In the description of the technique, the authors did not underline the importance that the excessive dilation of the left ventricle could have in the ventricular performance for the overcorrection of the PMs, nor the different anatomy of the papillary muscles that can deeply influence the surgical handling. Instead, we stressed two concepts: the importance of the anatomy of the papillary muscle and the degree of approximation based on the type of tethering and the degree of dilation of the ventricle. Rama et al. [62] considering the different anatomy of the papillary muscles simplified the technique and preliminary used a single stitch (Figure 3(b)).

**Figure 4:**
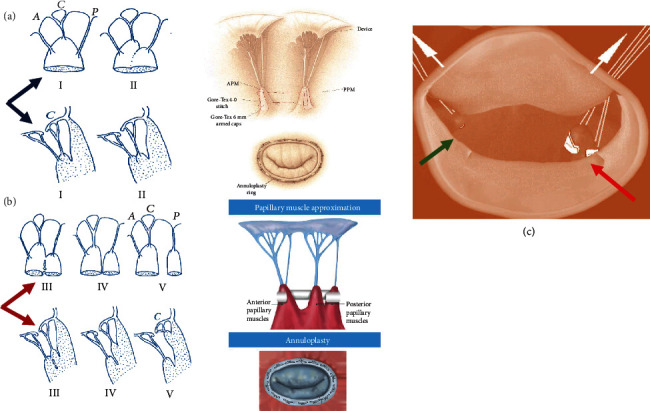
(a–c) Different surgical techniques of handling subvalvular apparatus. (a, b) Anatomy of the papillary muscles and corresponding handling surgical technique. (a) Type I, single uniform unit; type II, groove with two apexes (blue arrow). PMA using Goretex 4-0 stitch. (b) Type III, fenestrations with muscular bridges; type IV, complete separation in two adjacent heads; type V, complete separation with two distant heads (purple arrow). PMA using Goretex 4-0 prosthetic for PM sling. (c) Papillary muscle relocation through the fixation of the PMs to the trigone: APM (green arrow) and PPM (red arrow).

We use a 4-0 Goretex suture with pledget the anatomical for PM classified as I, II, and III and a 4-0 Goretex prosthesis for types IV and V [2–4]. Wakasa et al. reported, in patients with a transmural scar of the anterior LV wall, a complete side-by-side PMA through an anterior LV incision. The combined subvalvular repair and reconstruction of left ventricular wall was associated with concomitant MV annuloplasty [68] (Figures 4(a) and 4(b)).

*(2) Clinical Evidence*. In PMA RCT, we evaluated the effect of PMA on long-term outcomes in 96 patients who had moderate to severe mitral regurgitation. The 48 patients undergoing combined subvalvular repair and RMA had not significantly better 5-year survival rate than those (*n* = 48) whose IMR was managed with RMA alone (22.9% vs. 29.2%; HR 0.76; 95% confidence interval (CI), 0.35 to 1.68, *P* = 0.502). No statistically significant difference in moderate-to-severe MR recurrence was noted between the 2 groups after 2 years (RMA 13.2% vs. PMA 15.0%); However, at 5-year follow-up, we found a statistically significant difference between the proportion of surviving patients with recurrence of severe MR (RMA 23.5% vs. PMA 10.8%, *P* = 0.153). At 5-year follow-up after surgery in recipients of restrictive mitral annuloplasty plus subvalvular repair, there was an increase in further rehospitalization for heart failure that was lower to those who had received RMA alone (23.8% vs. 38%, *P* 0.136). During a follow-up period of 5 years, the incidence of MACCE was significantly reduced in the PMA group in the last year of follow-up (HR 0.10, 95% confidence interval (CI) 0.02 to 0.49, *P* = 0.004) [9, 23] (Table 5).

In another report by Wakasa et al. [68], 90 patients with IMR were studied. 30 patients received annuloplasty alone while 60 patients had combined subvalvular repair without (*n* = 26) or with (*n* = 34) left ventriculoplasty. During a median follow-up period of 3.4 years, the linearized mortality rate (all-cause and cardiac-related mortality) was comparable in patients with combined surgery versus isolated RMA (*P* = 0.61 and 0.92). The subvalvular repair without LV reconstruction was used for 26 patients. This surgical strategy was independently associated with a reduced risk of grade ≥ 2+ MR recurrence compared to subvalvular repair plus left ventriculoplasty or RMA alone (*P* = 0.09) (Table 5).

In a study from Iran et al. [8], the subvalvular mitral-valve repair was compared with RMA alone 100 consecutive patients (74% ischemic DCM and 26 nonischemic DMC) who were followed for a mean of 40.8 months. There was significant freedom from recurrence of 3+ to 4+ MR for the patients who underwent subvalvular mitral-valve repair as compared with those who underwent RMA alone both in ischemic DMC than in nonischemic DMC (PMA 3.4% vs. RMA ischemic DMC 8% vs. RMA nonischemic DMC 11%, *P* = 0.428). At the final follow-up, the New York Heart Association (NYHA) function class was 1.57 ± 0.62 in the annuloplasty group and 1.45 ± 0.57 in the combination group; there was no significant difference in NYHA function class between the first and final follow-up (*P* > 0.05) (Table 5).

Recently, the pivotal role of IPMD has been shown in a report from the Osaka Rosai Hospital [40]. The authors clarified the association between left ventricular (LV) function, the severity of mitral regurgitation (MR), and leaflet tethering parameters after RMA. 44 patients who underwent an isolated primary operation with RMA, between 2004 and 2015, were studied. During a median follow-up period of 66 months, the LV function, anterior and posterior PM tethering distance, anterior leaflet angle, and IPMD improved for 33 patients. Change in IPMD (31 ± 6 to 25 ± 5 mm) and posterior PM tethering (37 ± 4 to 32 ± 4 mm) was independently associated with a reduced risk of MR recurrence (parameter estimate of 0.299 with standard error of 0.110; *P* = 0.013 and parameter estimate of -0.104 with standard error of 0.045; *P* = 0.035). Moreover, the IPMD change was independently associated with a change in LV end-systolic dimension (parameter estimate of 0.299 with standard error of 0.110; *P* = 0.013) resulting in a better improvement of IPMD that is linked with the favorable reverse remodeling [40] (Table 5).

*(3) Limitation*. Criticisms concerning the use of the PMA procedure are justified when we analyze the results in depth. In fact, at 5-year follow-up, we found 23.8% of patients who had a rehospitalization for heart failure and 27% of patients revealing moderate to severe mitral (preoperative EROA 41.0 ± 5.3 vs. a postoperative EROA 41.1 ± 1.1). The reasons for this adverse result can be partially explained by the fact that the clinical benefit of additional PM approximation is multifactorial. Although surgery of papillary muscles may seem appropriate in patients with dilated left ventricles, with large areas of scar tissue formation, dyskinesia, or a basal aneurysm, prospective trials on the use of subvalvular surgery of mitral valve are currently insufficient to highlight improvements in postoperative tethering among patients with LV lateral wall dysfunction, persistent LV dyskinesis, and severe alteration of LV sphericity as well as predominant lateral displacement of both leaflets due to symmetric tethering [9, 23, 34, 56].

In recipients with combined restrictive mitral annuloplasty and subvalvular repair who had severe and proportionate mitral regurgitation, the surgery may not directly improve the prognosis. In this 23.8% of patients, the ischemic cardiomyopathy is the leading disease often independent by the degree of mitral regurgitation, showing an adverse global and localized left ventricular reverse remodeling at the five-year follow-up (LVEDD 62.7 ± 3.4 vs. LVEDD 63.5 ± 2.4, mean change from baseline −6.4 ± 0.49, EROA 41.0 ± 5.3 vs.41.1 ± 1, 114, 15, 23). Finally, despite a few improvements in adverse reverse left ventricular remodeling (mean compared to baseline −6.5 ± 0.7 at 5 years), 2 women (5%) undergoing combined surgery required further surgical intervention between 30 days and 5 years. Women with IMR who have combined subvalvular surgery experienced higher mortality after MV surgery than men despite no significant differences in the degree of reverse LV remodeling between sexes. This result seems to coincide with the subanalysis of the female gender subgroup of the CTSN RCT [9, 23].

#### 7.1.2. Papillary Muscle Relocation

*(1) Clinical Use*. After the initial experimental experience of Liel-Cohen in 2000 [69], Kron et al. [60] firstly reported the systemic use of relocation of posteromedial papillary muscle for 18 patients who developed a transmural infarction of the inferior wall with moderate to severe IMR. Since then, the PM relocation has been applied in clinical practice.

Briefly, 3-0 Prolene suture is passed through twice through the fibrous portion of the posterior papillary muscle tip and brought down to mitral annulus just posterior to right the fibrous trigone, drawing the PMPM tip closer to the annulus. The final position of the tip of the posterior papillary muscle was considered by determining the point at which the coaptation of the leaflets in the plane of the mitral annulus occurs.

Recently, Harmel et al. [10] used an alternative strategy for the relocation of posteromedial papillary muscle based on the following steps: (1) standardized apico-lateral realignment of both papillary muscles, (2) fixation of both papillary muscles' sutures on the posterior side of annuloplasty ring, and (3) systematic application in a three-dimensional endoscopic mini-thoracotomy setting (Table 5 and Figures 3(a) and 4(c)).

*(2) Clinical Evidence*. The pivotal series reported no short-term failures in two months, and the recipients of the relocation did not require the need for a new intervention with the subvalvular option [70, 71]. One propensity-matched study included 110 patients with follow-up duration exceeding a mean of 48 months and reported no difference in overall 5-year freedom from cardiac-related death rate in the papillary muscle relocation group compared to isolated restrictive mitral annuloplasty group (90.9% ± 1.8% and 89% ± 1.6% (*P* = 0.82)). Compared with the RMA, the subvalvular repair has a better 5-year freedom from cardiac-related event rate (83% ± 2.1% and 65.4% ± 1.2% (*P* < 0.001)). Only 1 RCT studied 101 patients comparing the combined papillary muscle relocation and restrictive mitral annuloplasty with RMA alone. During a maximum follow-up period of 1 year, the 51 patients undergoing subvalvular surgery had a significantly better 1-year survival rate than those whose ischemic mitral regurgitation was managed with isolated RMA (0% vs. 1 0% (*P* = 0.025)) and a significant tendency to better event-free MR recurrence (98% vs. 86.7% (*P* = 0.045)) [70, 71] (Table 5).

*(3) Limitation*. Very few candidates with postero-basal myocardial infarction and asymmetric tethering have contraindications to PM relocation. The technique has a low incidence of severe complications and a good capacity for maintaining the normal three-dimensional relationship between the posterior papillary muscle tip, leaflets, and the annulus. Importantly is that to minimize mitral valve tenting, the relocation of both PMPM is indicated [60, 70]. Although one head of the anterolateral PM can be transferred; however, with regard to the transfer of posteromedial PM, it is very important to consider the morphology of the PM characterized by the different anatomy identifiable in types III, IV, and V. So, it is crucial to relocate both heads of the PMPM, because from the more anterior head of PM emerges the chordae destined to the anterior leaflet and responsible for the seagull sign and respective tenting. Instead, from the more posterior head of PMPM derives the chordae for P2 and P3 scallops [60, 70, 72] (Figure 4(c)).

The biomechanical profile of the subvalvular repair has now been extensively studied, and the use of papillary muscle approximation or papillary muscle relocation does not increase perioperative risk. Furthermore, unlike PM approximation, PM relocation could result in potential adverse biomechanical effects. The latter does not address the multidirectional displacement and migration of the PM resulting in increased tension at the level of the posterior trigone and papillary muscle [35–37, 72–79]. Watanabe et al. [73] showed that the relocation of the PM might be associated with a restrictive effect on the MV if directed only to the posterior leaflet as inducing a tilting effect on the posterior annulus and augmenting its posterior tethering. To this extent, in the largest series of Fattouch and colleagues, the PM relocation was effective only in association with a nonrestrictive annuloplasty [70, 71].

#### 7.1.3. Ring Plus String Procedure

Introduced in mitral valve surgery in the 2000s [61], the ring and string procedure combines the use of RMA ring (RING) with papillary muscle repositioning (STRING). Concerns over the degree of the development of recurrent MR, due to LV remodeling for which TH is one of the more easily determined quantitative parameters, have been reduced after the demonstration of TH exceeding 10 mm. In these circumstances, almost all patients develop recurrent MR with the absence of reverse remodeling. There is a general agreement of adding papillary muscle repositioning to restrictive mitral annuloplasty indicated in patients with secondary MR > grade 3 and TH > 10 mm [34, 35, 56, 58, 59] (Tables 2–4).

*(1) Clinical Use*. Restrictive mitral annuloplasty is performed using an undersized ring through the choice of 1 to 2 sizes in relation to the intertrigonal distance. Firstly, a horizontal aortotomy is executed, and a double-armed Teflon pledgeted 4-0 expanded polytetrafluoroethylene (e-PTFE-Goretex) suture (STRING) is going through the head of the papillary muscle. Secondly, the stitch is passed from the LV cavity through the aorto-mitral continuity below the commissure between the noncoronary and left coronary aortic cusps and then exteriorized. In patients with ischemic MR due to asymmetric tethering, local LV remodeling (infero-basal scar tissue formation), the use of a single string for the PMPM is enough. In patients with nonischemic cardiomyopathy or ischemic MR characterized by global LV remodeling, the use of 2 strings, one for each papillary muscle, is more suitable [61].

## 8. Procedures Targeting Mitral Valve Leaflets

### 8.1. Edge-to-Edge Procedure Theory and Indication

The theoretical base for the surgical technique of the edge-to-edge leaflet coaptation was described by Alfieri et al. who showed that the creation of a double orifice mitral valve confers a prompt decrease in the severity of MR [80]. The benefit associated with the use of the edge-to-edge procedure in association with RMA is because the handling of MV is addressed directly to the site of the regurgitant jet. Thereby, suturing the edges of the MV leaflets together at the site of regurgitation may improve the durability of MV repair and prevent MR recurrence. The improved result using the Alfieri procedure is almost certainly due to the fact that it guarantees an early closing of the MV which is compromised by the reduction of the closing forces for the decrease of systolic tension [81]. Normally, the subvalvular apparatus exerts vertical tension to prevent prolapse during systole, which is disrupted with ventricular remodeling. Therefore, when the leaflets are anchored together, they could exert an upward tension on the chordae tendineae, on the papillary muscles, and on the adjacent LV wall. This action can potentially counteract the progression of LV adverse remodeling [74, 77].

The edge-to-edge operation is considered for patients with MR secondary to ventricular disease, due to nonischemic or ischemic remodeling. Since there are similarities in the pathophysiology of the geometrical alteration between two phenotypes, the procedure is applicable to both conditions considering the grade of coaptation depth or tenting height (TH). The best results the Alfieri procedure yields are related to patient selection. Therefore, during the handling of the mitral valve, the evaluation of the distance from the annular plane of the MV to the leaflet coaptation point should be considered. In fact, this parameter represents the degree of tethering of the mitral leaflet, regardless of the LV function and tethering shape [54, 55, 82, 83]. The use of edge-to-edge operation in combination with RMA is preferred in patients with TH > 10 mm [34, 56].

#### 8.1.1. Clinical Use

Patients who are scheduled to receive the edge-to-edge operation should undergo transthoracic echocardiography to assess the mechanism and severity of mitral regurgitation for choosing the site of the approximating stitch. The phenotype identified in symmetric tethering is characterized by a central jet located between A2 and P2 scallops of mitral valve leading for a central edge-to-edge by creating a double-orifice MV configuration. When the regurgitant jet is located at the posterior commissure, as in some cases of ischemic MR, a commissural edge-to-edge suture is applied, resulting in a single orifice MV with a relatively smaller area. The length of the suture is always kept as short as possible to minimize the risk of postoperative MV stenosis: in most patients between a few millimeters and 1 cm. A complete rigid or semirigid prosthetic ring is invariably implanted and is usually 1 or 2 sizes smaller than the anterior leaflet surface [80, 84–86].

#### 8.1.2. Clinical Evidence

The survival benefits associated with the use of combined RMA with edge-to-edge procedure over RMA alone were established in a landmark paper from the San Raffaele University Hospital almost 15 years ago. The improved outcome using the edge-to-edge suture is almost certainly due to its superior long-term freedom from MR recurrence. Although several studies have reported substantially disappointing results using an Alfieri stitch, however, these reports are focused on the edge-to-edge procedure without concomitant RMA. Pioneer series evaluated the effect of MV surgery in 77 patients who had moderate to severe idiopathic (*n* = 26) or ischemic (*n* = 51) functional mitral regurgitation and with follow-up duration exceeding a mean of 18.4 months. RMA combined with the edge-to-edge procedure was used in 54 recipients (TH > 10 mm) while isolated RMA in 23 (TH < 10 mm). The 54 patients whose mitral regurgitation was managed conservatively using associated edge to edge had a significantly better 2.7-year survival rate than those who underwent isolated RMA (91.4 ± 4.1% vs. 89.2 ± 7.2%, *P* = 0.9). Among patients who had MR recurrence ≥3 + /4+, the rate of 3.7% received a concomitant edge-to-edge repair while 21.7% is undergoing isolated RMA (*P* = 0.03). Although freedom from repair was 95 ± 3.4% and 77 ± 12.1%, respectively (*P* = 0.04), however, the absence of the edge-to-edge was the only predictor of repair failure (*P* = 0.03). In both groups, LV end-diastolic dimensions decreased (67 to 58 mm after RMA and 68 to 62 mm after RMA with edge-to-edge procedure), and NYHA functional class improved after surgery. Of note in patients undergoing concomitant mitral annuloplasty with edge-to-edge repair, larger rings were used to avoid stenosis during the edge-to-edge suture [87].

In another report, 54 patients with moderate to severe functional mitral regurgitation (FMR) due to nonischemic idiopathic dilated cardiomyopathy were studied. During a median follow-up period of 4.2 years, the use of the edge-to-edge technique, despite favoring reverse LV remodeling, was not associated with a reduced risk of death (77.7 ± 9.9% associated edge-to-edge technique to RMA and 87.7 ± 5.8% isolated RMA (*P* = 0.5); univariate HR 2.3; 95% CI, 0.9 to 6.1; *P* = 0.01; multivariate HR 1.8; 95% CI, 0.6–4.8; *P* = 0.2) [88].

#### 8.1.3. Limitation

Echocardiography-based follow-up studies have identified that the edge-to-edge procedure may determine a reduction in the area of the MV, leading to mitral valve stenosis which can be clinically relevant. We have used this procedure in patients with ring sizes greater than 36 mm and with a tenting height > 10 mm. Usually, we used a 18 mm Hegar probe number to measure the anterior commissure and 17 mm for the posterior one, respectively [86]. The careful choice of the annuloplasty ring size (downsizing by 1 measure) is mandatory in order to avoid a significant MV stenosis [80, 87]. We have not used edge-to-edge procedures in severe dilation of the left ventricle (LVEDD > 65 mm) marked by extremely advanced LV remodeling [56].

### 8.2. Anterior Leaflet Enlargement and Cutting Secondary Chordae

Anterior leaflet (AL) enlargement was initially described in the French correction to relieve the restricted leaflets' motion in systole and diastole in patients with mitral valve rheumatic disease (Carpentier type IIIa) [3]. Subsequently, it was successfully applied to counteract the leaflet coaptation defect due to tethering for the displacement of the posterior papillary muscle [89–91]. The AL enlargement is achieved by means of the use of glutaraldehyde-fixed autologous or bovine pericardial patches.

We use the autologous pericardium patches in maintaining an optimal quality of life free from long-term anticoagulant treatment in patients with Carpentier type IIIa and IIIb insufficiency [91–93]. We recorded 90 patients (70 females, 20 males) in which we tailored the use of anterior mitral leaflet augmentation with autologous pericardium fixed with glutaraldehyde to etiology of patients. The mode of dysfunction was pure mitral insufficiency in 71 cases (78.9%) and insufficiency associated with stenosis in 19 cases (21.1%). In our experience, we reported that despite the use of an undersized annuloplasty, persistence or recurrence of mitral regurgitation can occur due to the severity of tissue retraction preventing leaflet coaptation [91–93].

The anterior leaflet was measured using a ring sizer, and if the degree of leaflet retraction did not safely allow the downsizing of the annuloplasty ring by two sizes (i.e., risk of stenosis), the anterior leaflet augmentation technique was chosen [31]. To avoid any damage to the pericardial patch suture, all stitches devoted to prosthetic ring annuloplasty (2-0 Ethibond (Ethicon Inc, Piscataway, NJ)) were placed before anterior leaflet augmentation. Mitral annuloplasty was performed with a flexible ring in 65 cases, and we used a rigid ring in 16 cases; 9 patients were managed without the use of annuloplasty [91–93].

The technique of anterior leaflet augmentation has been shown to be safe and reproducible, and the enlargement involved not only the anterior leaflet (Carpentier types IIIa and IIIb) but also the commissural areas (Carpentier type IIIa). The procedure may be coupled with cutting secondary chordae to reduce the effect of leaflet tethering cause by the displacement of PMs [94–99].

AL enlargement is effective when the TH does not exceed 8 mm and with LV chambers not very enlarged (LVESD < 50 mm and LVEDD < 60 mm) in which a favorable ventricular remodeling is implemented by optimal CABG operation. In the case of an increased risk of persistent tethering, the procedure is not indicated [33, 34, 56].

The use of cutting secondary chords by performing the “chordal cutting” has been proposed as a surgical handling of mitral valve for decreasing leaflet tethering and mitral regurgitation in patients with ischemic mitral regurgitation [94–99]. The surgical benefit of chordal cutting coupled to restrictive mitral annuloplasty has been recorded in patients with predominant cause of chronic secondary due to apical displacement leading to exacerbate tethering of the MV leaflets [96–99]. The procedure, which may be achieved with also through aortotomy [97], was aimed at preventing central coaptation during systole [94–99] (Figure 3(a)).

Boger et al. [95] reported echocardiographic and clinical advantage in patients who received chordal-cutting mitral valve repair (*n* = 43) compared to those who were managed with the use of conventional restrictive mitral valve repair. In patients who underwent the chordal cutting procedure, authors recorded a greater reduction in tent area than those who received RMA alone (53 ± 3% vs. 41 ± 3%; *P* = 0.01). Surely in recipients of chordal-cutting, a greater mobility of the anterior leaflet occurred, leading for a reduction in the distance between the free edge of the anterior mitral valve leaflet and the posterior left ventricular wall (24 ± 3% vs. 11 ± 4%; *P* = 0.01).

The major finding of Boger's study revealed that patients who received RMA alone had more recurrent mitral regurgitation during 2 years of follow-up as demonstrated with univariate (37% vs. 15%; *P* = 0.03) and multivariate Cox regression analysis (*P* = 0.03). In addition, the use of chordal cutting did not adversely affect postoperative left ventricular ejection fraction (10% ± 5% relative increase in left ventricular ejection fraction vs. 11% ± 6% in the control group; *P* = 0.9).

In patients who have a very dilated left ventricular chamber, the use of chordal cutting is preferable coupled to anterior leaflet enlargement [94].

### 8.3. Transcatheter Heart Valve Therapy

Landmark studies have reported that the use of transcatheter heart valve therapy (THVT) has proven safety and effectiveness in patients with symptomatic moderate to severe secondary MR [4, 5]. This type of mechanical intervention is based on the percutaneous approach and on the use of the edge-to-edge procedure [87, 88] with the fitting of 2/3 metal clips (i.e., MitraClip procedure, Abbott Vascular, Santa Clara, CA, USA).

In COAPT [4] and MITRA-FR [5] RCTs, the use of THVT recorded better survival than optimal medical therapy alone (GDMT) in patients with advanced ischemic cardiomyopathy, marked reduction of symptoms associated with heart failure-associated symptoms, and significant improvement in quality of life.

The detailed analysis of the results of the RCTs however revealed two distinct phenotypes of patients suggesting the possibility of categorizing proportionate and disproportionate patients thus highlighting an inhomogeneous response after transcatheter mitral valve repair [100, 101].

The proportionate phenotype [100, 101] in the MITRA-FR [5] study included patients with moderate or moderate to severe MR and severely dilated left ventricular chamber. At a maximum follow-up of 12 months, the use of MitraClip recorded no differences in the rate of death or unplanned hospitalization for heart failure compared to GDMT. Conversely, in patients enrolled in the COAPT [4] RCT and included as a disproportionate phenotype [100, 101], severe secondary MR was associated with less severe left ventricular dilation with better preserved ventricular function. Mitral valve repair with the MitraClip procedure resulted in a lower rate of rehospitalization for heart failure and all-cause mortality within 24 months significantly greater than medical therapy alone.

The unevenness of the results suggests great caution in extending THVT to categories of patients indicated as intermediate and low risk [102–104].

### 8.4. Left Ventricular Assist Device: When and How

Existing evidence has shown that secondary IMR is a serious disease, and patients with worsening left ventricular dysfunction may not respond to medical therapy and conventional mechanical intervention procedures, thus showing a very poor prognosis.

Patients who have worse left ventricular remodeling that can lead to further deterioration of LV function and recurrence of MR have an unfavorable pathological substrate. For these patients, the use of the transcatheter approach, while maintaining the advantage of avoiding the perioperative risks of the standard intervention, may sometimes not be decisive [4, 5].

The results of the two recent randomized studies MITRA-FR [5] and COAPT [4] reported inconsistent results regarding the efficacy of the MitraClip compared to the GDMT. For patients with more severe left ventricular dysfunction who fall within the pathophysiological condition of proportionate, the multidisciplinary Heart Team should be considered to receive LVAD implantation as a viable alternative.

Procedures for using LVAD have improved over the past decade, and the survival of patients benefiting from the LVAD implant has steadily improved in parallel with the technological advances of the armamentarium platform that supports the LVAD implant procedure. The work of the multidisciplinary Heart Team has improved patient selection, perioperative management, and outpatient treatment.

Patients with severe end-stage heart failure who were managed with the use of ventricular assist devices demonstrated better outcomes than those who received a GDMT, as evidenced by the dramatic improvement in life expectancy [105, 106].

Today, the results after LVAD implantation show survival that reaches approximately 75% at 1 year [107]. Sometimes, a combination of LVAD implantation and mitral valve repair is indicated [108]. In most centers, this associated procedure is not used because mitral regurgitation is minimal during LVAD operation due to the continuous aspiration of the device. It is important to note that mitral regurgitation occurs in mild to moderate/severe degrees during weaning from the device.

Patients with secondary IMR who have received LVAD are at risk of developing thromboembolic events related to anticoagulation, bleeding, and infection. LVAD implantation should be considered prior to deterioration of right ventricular function [109].

## 9. Discussion

The unanimous and shared strategy for the optimal treatment of patients with secondary IMR has not yet been achieved. The first-rate management for these patients remains drug therapy and combined resynchronization with device implantation as recommended by the guidelines [102–104]. Patients who do not obtain a benefit despite optimal medical treatment should be directed to referral centers specialized in the treatment of heart failure [102–104].

**Figure 5:**
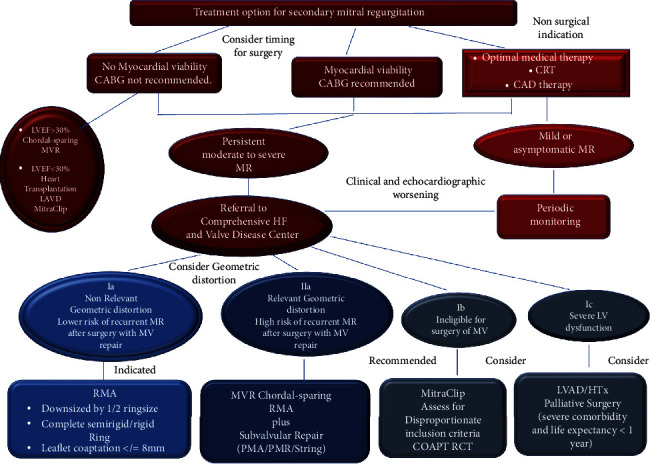
This flowchart summarizes the current options to treat ischemic functional mitral regurgitation, based on the most recent and relevant literature.

The management of patients with secondary mitral regurgitation must be entrusted to a multidisciplinary Heart Team, whose work is carried out by specialists in different fields: heart failure, interventional cardiology, cardiology of arrhythmias, cardiac surgery, and treatment of heart failure. The coordinated work of the team is committed to orienting the patient towards the best possible treatment option [102–104] (Figure 5).

Patients with disease progression and life expectancy < 1 year and with severe comorbidities should be directed to palliative therapy [102–104].

The clinical presentation of the patient may be variable and not limited solely to FMR surgery. So, a concomitant procedure such as CABG operation, treatment of severe tricuspid regurgitation, and direct mechanical intervention to cure cardiac arrhythmia may be necessary.

Mitral valve repair has long been considered the primary option for clinical benefits supported primarily by retrospective cohort studies. A veil of uncertainty about the use of MV repair has been lifted by the evidence reported in the RCTs [1, 2, 47] of the CTSNet which suggests MV replacement in case of severe mitral regurgitation [2].

The MV repair option is not indicated for the subgroup of patients who are at high risk for reverse left ventricular remodeling and/or recurrent MR. For patients with a low degree of geometric distortion (LVEDD < 60 mm; LVESD < 55 mm; TH between 5 mm and 8 mm), RMA with or without anterior leaflet augmentation is indicated if combined with complete revascularization of the ischemic myocardium in the presence of nonextensive postscarring infarct. Generally, the extension of the infarct zone into the lateral territory, perfused by the circumflex artery, leaves more extensive scars thus responsible for an accentuated apical tethering. In these patients, the TH exceeds 8 mm, reducing the chance of RMA success. A combined RMA procedure with subvalvular repair is recommended in patients with greater geometric distortion of the LV chambers (LVEDD > 60 mm; LVESD > 55 mm; TH exceeds 8 mm). In this circumstance, the other procedures that can be considered in addition to the RMA are the edge-to-edge procedure and RING + STRING. Although these techniques may improve outcomes in terms of no recurrence of IMR and reverse LV remodeling, however, they have not been widely used leading to scarce evidence as reported in long-term follow-up.

For patients with moderate S-MVR, the use of an RMA associated with CABG operation is indicated in case of extensive postinfarct scars also in combination with a subvalvular repair if the geometric distortion of the ventricle is accentuated as in the apical tethering of the leaflets [9, 23, 24, 110]. The use of CABG operation alone is recommended in patients with ischemic S-MVR and favorable remodeling of the LV [111]. The most difficult categories of patients to treat are those indicated by Grayburn as proportionate in which the MR is associated with a severely dilated left ventricle, with a worse left ventricular function and remodeling [95, 96, 112]. For this category of patients, which mainly suffering from nonischemic S-MVR due to cardiomyopathy, even the use of the transcatheter mitral valve therapy does not seem to lead to good results for the higher tendency to the progression of the cardiomyopathic disease [5, 95, 96, 112].

The use of the MitraClip procedure is recommended in patients with disproportionate secondary mitral regurgitation in which severe MR is coupled with nonseverely dilated left ventricular chambers [95, 96]. Although this new framework is a very attractive proposal, however, it does not find unanimous consensus and requires further deep investigation involving transcatheter heart valve therapy [113–127].

## 10. Conclusion

The use of subvalvular repair associated with RMA is the new procedural key to achieve efficacy in the treatment of secondary mitral regurgitation in both patients with asymmetrical tethering and in those with symmetrical tethering. Restrictive mitral annuloplasty associated with cutting chordal procedure and/or to anterior mitral leaflet enlargement may be suitable for patients with moderate geometrical abnormalities of mitral valve and with nonsevere left ventricular dilatation.

## Figures and Tables

**Table 1:** PRISMA Checklist item.

Section and topic	Item #	Table 1 checklist item	Location where item is reported
*Title*			
Title	1	Identify the report as a systematic review.	Title
*Abstract*			
Abstract	2	See the PRISMA 2020 for Abstracts checklist.	Abstract
*Introduction*			
Rationale	3	Describe the rationale for the review in the context of existing knowledge.	Introduction
Objectives	4	Provide an explicit statement of the objective(s) or question(s) the review addresses.	Introduction
*Methods*			
Eligibility criteria	5	Specify the inclusion and exclusion criteria for the review and how studies were grouped for the syntheses.	Methods
Information sources	6	Specify all databases, registers, websites, organisations, reference lists, and other sources searched or consulted to identify studies. Specify the date when each source was last searched or consulted.	Methods
Search strategy	7	Present the full search strategies for all databases, registers, and websites, including any filters and limits used.	Methods
Selection process	8	Specify the methods used to decide whether a study met the inclusion criteria of the review, including how many reviewers screened each record and each report retrieved, whether they worked independently, and if applicable, details of automation tools used in the process.	Methods
Data collection process	9	Specify the methods used to collect data from reports, including how many reviewers collected data from each report, whether they worked independently, any processes for obtaining or confirming data from study investigators, and if applicable, details of automation tools used in the process.	Methods
Data items	10a	List and define all outcomes for which data were sought. Specify whether all results that were compatible with each outcome domain in each study were sought (e.g., for all measures, time points, and analyses), and if not, the methods used to decide which results to collect.	Methods
	10b	List and define all other variables for which data were sought (e.g., participant and intervention characteristics and funding sources). Describe any assumptions made about any missing or unclear information.	Methods
Study risk of bias assessment	11	Specify the methods used to assess risk of bias in the included studies, including details of the tool(s) used, how many reviewers assessed each study, and whether they worked independently, and if applicable, details of automation tools used in the process.	Methods
Effect measures	12	Specify for each outcome the effect measure(s) (e.g., risk ratio and mean difference) used in the synthesis or presentation of results.	Tables 1 and 2
Synthesis methods	13a	Describe the processes used to decide which studies were eligible for each synthesis (e.g., tabulating the study intervention characteristics and comparing against the planned groups for each synthesis (item #5)).	Methods
	13b	Describe any methods required to prepare the data for presentation or synthesis, such as handling of missing summary statistics, or data conversions.	N/A
	13c	Describe any methods used to tabulate or visually display results of individual studies and syntheses.	Methods
	13d	Describe any methods used to synthesize results and provide a rationale for the choice(s). If meta-analysis was performed, describe the model(s), method(s) to identify the presence and extent of statistical heterogeneity, and software package(s) used.	N/A
	13e	Describe any methods used to explore possible causes of heterogeneity among study results (e.g., subgroup analysis and metaregression).	N/A
	13f	Describe any sensitivity analyses conducted to assess robustness of the synthesized results.	N/A
Reporting bias assessment	14	Describe any methods used to assess risk of bias due to missing results in a synthesis (arising from reporting biases).	Methods
Certainty assessment	15	Describe any methods used to assess certainty (or confidence) in the body of evidence for an outcome.	Tables 1 and 2
*Results*			
Study selection	16a	Describe the results of the search and selection process, from the number of records identified in the search to the number of studies included in the review, ideally using a flow diagram.	Figure 1
	16b	Cite studies that might appear to meet the inclusion criteria, but which were excluded, and explain why they were excluded.	Figure 1
Study characteristics	17	Cite each included study and present its characteristics.	Tables 1 and 2
Risk of bias in studies	18	Present assessments of risk of bias for each included study.	N/A
Results of individual studies	19	For all outcomes, present, for each study: (a) summary statistics for each group (where appropriate) and (b) an effect estimate and its precision (e.g., confidence/credible interval), ideally using structured tables or plots.	Tables 1 and 2
Results of syntheses	20a	For each synthesis, briefly summarize the characteristics and risk of bias among contributing studies.	N/A
	20b	Present results of all statistical syntheses conducted. If meta-analysis was done, present for each the summary estimate and its precision (e.g., confidence/credible interval) and measures of statistical heterogeneity. If comparing groups, describe the direction of the effect.	N/A
	20c	Present results of all investigations of possible causes of heterogeneity among study results.	N/A
	20d	Present results of all sensitivity analyses conducted to assess the robustness of the synthesized results.	N/A
Reporting biases	21	Present assessments of risk of bias due to missing results (arising from reporting biases) for each synthesis assessed.	N/A
Certainty of evidence	22	Present assessments of certainty (or confidence) in the body of evidence for each outcome assessed.	Tables 1 and 2
*Discussion*			
Discussion	23a	Provide a general interpretation of the results in the context of other evidence.	Discussion
	23b	Discuss any limitations of the evidence included in the review.	Discussion
	23c	Discuss any limitations of the review processes used.	Discussion
	23d	Discuss implications of the results for practice, policy, and future research.	Discussion
*Other information*			
Registration and protocol	24a	Provide registration information for the review, including register name and registration number, or state that the review was not registered.	N/A
	24b	Indicate where the review protocol can be accessed, or state that a protocol was not prepared.	N/A
	24c	Describe and explain any amendments to information provided at registration or in the protocol.	N/A
Support	25	Describe sources of financial or nonfinancial support for the review and the role of the funders or sponsors in the review.	N/A
Competing interests	26	Declare any competing interests of review authors.	Title page
Availability of data, code, and other materials	27	Report which of the following are publicly available and where they can be found: template data collection forms, data extracted from included studies, data used for all analyses, analytic code, and any other materials used in the review.	N/A

**Table 2:** Outcomes of S-MVR surgery.

First author (ref. #)	Number of patients	Follow-up (yrs)	Type of surgery	Main findings
Goldstein et al. [2]	251	2	MVRpl/RMA 125/126	Higher rate of MR recurrence in RMA
Lorusso et al. [39]	488	8	MVRpl/RMA 244/244	Higher rate of MR recurrence in RMA
Magne et al. [20]	370	12	MVRpl/RMA 184/185	Similar rate of MR recurrence
Micovic et al. [13]	138	7	MVRpl/RMA 52/86	Better survival in RMA
Milano et al. [14]	522	12	MVRpl/RMA 106/416	Lower rate of 30-day mortality in RMA
Silbermann et al. [15]	52	3,2	MVRpl/RMA 14/38	Similar rate of survival. Better improvement in NYHA for RMA
Thourani et al. [21]	1250	10	MVRpl/RMA 625/625	Better 30 days and 10 yrs survival in RMA
McGee et al. [18]	585	5	RMA	After 1 yrs 3+ or 4+ MV regurgitation increased (*P* < .0001)
Yan et al. [12]	36	1	MVRpl-cp/MVRpl-pp 21/15	Significant early advantage with MV Sparing procedure
Natsuaki et al. [16]	28	30 days	MVRpl-cp/MVRpl-pp/RMA 15/13/15	Significant early advantage with MV Sparing procedure
Rozich et al. [22]	15	7/10 days	MVRpl-cp/MVRpl-pp 8/7	MVRpl-cp smaller LV size and LV function
David et al. [11]	17	24 months	MVRpl-cp/MVRpl-pp/RMA 6/6/5	Better improvement of LV function. Significant benefit in preservation of chordae attachment to leaflet and PMs

LV: left ventricle; MR: mitral regurgitation; MVRpl: mitral valve replacement; MVRpl-cp: mitral valve replacement complete preservation; MVRpl-pp: partial preservation; PMs: papillary muscles; RMA: restrictive mitral annuloplasty; S-MVR: secondary mitral valve regurgitation surgery. See text for other abbreviations.

**Table 3:** Studies comparing RMA ring undersized.

First author (ref. #)	Number of patients	UMA (1 vs. 2 sizes)	Type of surgery	Main findings
Furukawa et al. [8]	14	2 UMA	RMA	Higher rate of MR recurrence in dilated LV
Harmel et al. [10]	50	1 UMA	RMA	Higher rate of MR recurrence
Kainuma et al. [30]	44	2 UMA	RMA	Few rate of MR recurrence LVESD < 50 mm Better improvement of IPMD in favorable LV remodeling
Nappi et al. [34]	48	2 UMA	RMA	Few rate of MR recurrence LVESD < 50 mm
Capoulade et al. [32]	126	1 UMA	RMA	Few rate of MR recurrence LVESD < 50 mm
Nappi et al. [30]	22	2 UMA	RMA-sr/RMA-dr 11/11	Few rate of MR recurrence in RMA-dr
Kron et al. [55]	126	1 UMA	RMA	Higher rate of MR recurrence and mitral valve leaflet tethering
Kron et al. [28]	479	1 UMA	RMA-c/RMA-p 209/270	Higher rate of MR recurrence in RMA-p
Fattouch et al. [12]	55	1 UMA	RMA	Higher rate of MR recurrence in RMA
Langer et al. [61]	30	2 UMA	RMA	Higher rate of leaflet tethering in dilated LV
McGee et al. [18]	585	1UMA	RMA-rr/RMA-fr 396/122	After 1 yrs 3+ or 4+ MV regurgitation increased (*P* < 0.0001). Similar results in rr vs. fr

RMA: restrictive mitral annuloplasty; c: complete; dr: double row; fr: flexible ring; p: partial; rr: rigid ring; sr: single row; UMA: undersized mitral annuloplasty. See text for other abbreviations.

**Table 4:** Receiver-operating characteristic curve analyses, discriminative cutoff values, and diagnostic models associated with recurrent moderate-to-severe mitral regurgitation after RMA [8, 10, 12, 14, 18, 28, 30–34, 38, 40–42, 55, 56].

Valvular parameters	Ventricular parameters included PASP	Symmetric MV tethering pattern diagnostic models†
2.5 cm^2^ ≤ MV tenting area ≥ 3.1 cm^2^ or 2.6 cm^2^ ≤ MV tenting area ≥ 3.2 cm^2^	LV end-systolic volume > 145 ml or LVESV/BSA/ml/m^2^65	MV tenting area ≥ 3.1 cm^2^ + symmetric MV tethering
10 ≤ tenting height ≥ 12 mm	64 mm ≤ LVEDD ≥ 65 mm	MV tenting area ≥ 3.1 cm^2^ + LVEDD ≥ 64 mm
Posterior leaflet angle ≥ 45°	51 mm ≤ LVESD ≥ 54 mm	MV tenting area ≥ 3.1 cm^2^ + LVESD ≥ 54 mm
Posterior leaflet tethering distance ≥ 40 mm	PASP51 ≥ mm Hg	MV tenting area ≥ 3.1 cm^2^ + PASP ≥ 51 mm Hg
Mitral annulus diameter ≥ 37 mm	Interpapillary muscle distance > 20 mm	
MR grade ≥ 3.5	Systolic sphericity index > 0.7	
Central or complex regurgitant jet	Myocardial performance index > 0.9	
	Wall motion score index > 1.5	
	Presence of a basal aneurysm/dyskinesis	
	Diastolic dysfunction (restrictive filling pattern)	

**Table 5:** Studies comparing subvalvular repair plus RMA vs. RMA alone.

First author (ref. #)	Number of patients	Follow-up (yrs)	Type of surgery	Main findings
Furukawa et al. [8]	36	5	RMA plus SVR/RMA 22/14	Few rate of MR recurrence in SVR. Improvement in LV remodeling
Harmel et al. [10]	50	1	RMA plus SVR/RMA 50/50	Higher survival rate in SVR
Nappi et al. [9]	96	5	RMA plus SVR/RMA 48/48	Better improvement LV remodeling and MV geometry in SVR
Wakasa et al. [68]	90	6,4	RMA plus SVR/RMA 60/30	Better improvement LV remodeling and function in SVR and LV reconstruction
Roshanali et al. [71]	90	5	RMA plus SVR/RMA 60/30	Few rate of MR recurrence and better improvement in survival in SVR
Fattouch et al. [70]	110	5	RMA plus SVR/RMA 55/55	Few rate of MR recurrence in SVR
Hvass et al. [7]	37	7	SVR/RMA 37	Few rate of MR recurrence in SVR and significant improvement in leaflet tethering
Shudo et al. [40]	16	3,5	RMA plus SVR/RMA 15/11	Significant improvement in leaflet tethering in SVR
Langer et al. [61]	60	2	RMA plus SVR/RMA 30/30	Few rate of MR recurrence and reoperation in SVR. Significant improvement in leaflet tethering

RMA: restrictive mitral annuloplasty; SVR: subvalvular repair. See text for other abbreviation.

## Data Availability

The extracted data are included in the PRISMA Flow Diagram and in the PRISMA 2020 Checklist.
